# Inflammatory and immune markers associated with physical frailty syndrome: findings from Singapore longitudinal aging studies

**DOI:** 10.18632/oncotarget.8939

**Published:** 2016-04-22

**Authors:** Yanxia Lu, Crystal Tze Ying Tan, Ma Shwe Zin Nyunt, Esther Wing Hei Mok, Xavier Camous, Hassen Kared, Tamas Fulop, Liang Feng, Tze Pin Ng, Anis Larbi

**Affiliations:** ^1^ Department of Clinical Psychology and Psychiatry/School of Public Health, Zhejiang University College of Medicine, Hangzhou, China; ^2^ Singapore Immunology Network (SIgN), Agency for Science, Technology and Research (A*STAR), Singapore; ^3^ Gerontology Research Programme, Department of Psychological Medicine, Yong Loo Lin School of Medicine, National University of Singapore, Singapore; ^4^ Geriatrics Division, Department of Medicine, Research Center on Aging, University of Sherbrooke, Sherbrooke, Quebec, Canada; ^5^ Graduate Medical School, Duke-National University of Singapore, Singapore

**Keywords:** inflammation, immunosenescence, frailty risk, T cell subsets, B cells, Gerotarget

## Abstract

Chronic systematic inflammation and reduced immune system fitness are considered potential contributing factors to the development of age-related frailty, but the underlying mechanisms are poorly defined. This exploratory study aimed to identify frailty-related inflammatory markers and immunological phenotypes in a cohort of community-dwelling adults aged ≥ 55 years. Frailty was assessed using two models, a Frailty Index and a categorical phenotype, and correlated with levels of circulating immune biomarkers and markers of senescence in immune cell subsets. We identified eight serological biomarkers that were associated with frailty, including sgp130, IL-2Rα, I-309, MCP-1, BCA-1, RANTES, leptin, and IL-6R. Frailty Index was inversely predicted by the frequency of CD3+, CD45RA+, and central memory CD4 cells, and positively predicted by the loss of CD28 expression, especially in CD8+ T cells, while frailty status was predicted by the frequency of terminal effector CD8+ T cells. In γ/δ T cells, frailty was negatively associated with CD27, and positively associated with IFNγ+TNFα- secretion by γ/δ2+ cells and IFNγ-TNFα+ secretion by γ/δ2- cells. Increased numbers of exhausted and CD38+ B cells, as well as CD14+CD16+ inflammatory monocytes, were also identified as frailty-associated phenotypes. This pilot study supports an association between inflammation, cellular immunity, and the process of frailty. These findings have significance for the early identification of frailty using circulating biomarkers prior to clinical manifestations of severe functional decline in the elderly.

## INTRODUCTION

Frailty has been described as a nonspecific state of impaired strength, endurance, and balance; vulnerability to trauma and other stressors; and high risk for morbidity, disability, mortality and institutionalization [1]. The hematological, inflammatory, and neuro-endocrine systems, amongst others, are affected by age-related changes, resulting in a loss of muscle mass and strength (known as sarcopenia), increased susceptibility to disease or comorbidities, and general poor health [2–4]. However, the underlying pathophysiology of frailty is complex and incompletely understood.

Chronic inflammation is regarded as a major pathophysiological factor responsible for the progression of multiple chronic diseases, such as atherosclerosis, type 2 diabetes, Alzheimer's disease, and possibly, frailty. Studies of aging in mouse models have identified elevated levels of inflammatory markers, including macrophage inflammatory protein (MIP)-1α, MIP-1β, RANTES (Regulated upon Activation, Normal T-Cell Expressed and Secreted), and lymphotactin (Ltn) in freshly isolated CD4+ T cells from aged mice [5, 6]. Similarly, data from the Women's Health and Aging Studies (WHAS) (Baltimore, USA) [7] identified an association of frailty with high levels of IL-6, supporting an involvement of inflammatory mediators, although this study did not consider the effects of related factors in the IL-6 system, such as the levels of soluble IL-6 receptor and sgp130 [8].

An immune risk profile (IRP), identified in a population of Swedish elderly as CMV positivity with an inverted CD4:CD8 ratio and high CD8+CD28- frequency, was shown to predict mortality, suggesting a possible paradigm for an immune signature of frailty [9, 10]. Separate studies have reported similar changes in CD4 and CD8 T cell frequencies in frail individuals [11, 12]. A population-based study in the very old (85+ years) [13] observed an inverse relationship of memory/naïve CD8 T cell ratio with frailty, although this was in the opposite direction to that expected. Most research on immunosenescence has focused on changes in the α/β T cell compartment [14]; however, it is likely that other cell subsets are also altered during aging. The frequency of γ/δ T cells is thought to continuously decrease during aging [15], and their capacity for proliferation was found to be impaired in the elderly, while susceptibility to apoptosis was increased. The reduced frequency of γ/δ2+ T cell is thought to be related to a shift from naïve and central memory subsets (CCR7+CD27+) towards more differentiated phenotypes (CCR7+/−CD27+/−) [16]. The number and diversity of circulating B cells is also reduced in aging [17], which may be related to the decline in the numbers of germinal centers and B cell progenitors reported in human tonsils. It has been reported that aging does not affect dendritic cell (DC) function or phenotype [18, 19]; however, decreased frequencies of plasmacytoid DCs [20, 21] and CD14+CD16+ monocytes have been demonstrated in frail elderly populations [22].

Understanding the immune mechanisms underlying frailty is of great interest both for the prediction of remaining longevity and for the identification of potential prophylactic and/ or therapeutic immune rejuvenation strategies for the elderly. We undertook an extensive exploration of immune function in a cohort of community-dwelling older persons (aged 55 and above), characterized according to their level of frailty. High throughput Luminex technology was used to measure circulating levels of a wide range of cytokines, chemokines, and their receptors for the identification of biomarkers associated with the development of frailty. In addition, immune cell phenotypes were analyzed using multi-color flow cytometry to characterize the association of differentiation and senescence of various immune cells, including α/β T cells, γ/δ T cells, B cells, DCs, and monocytes, with frailty.

## RESULTS

### Frailty characterization and related functional change

**Figure 1:**
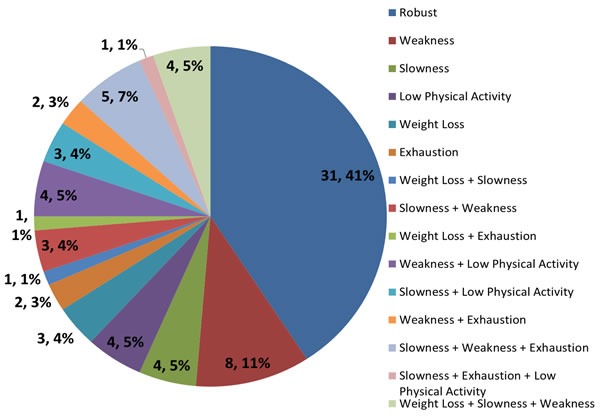

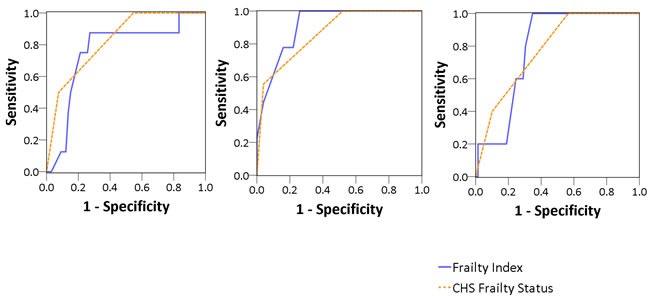
Frailty components and related functional change in a Chinese elderly population aged 55 years and over **A.** CHS frailty status and proportions of syndrome components in the elderly. The data are shown as n, %; **B.** Receiver operating curve analyses of the Frailty Index and the Fried frailty status predicting (from left to right) ADL-IADL dependency, lowest tertile of SF-12 physical health, and hospitalizations in the past year. CHS = Cardiovascular Health Study; ADL = Activities of Daily Living; IADL = Instrumental Activities of Daily Living; SF-12: 12-item Short Form Health Survey.

We measured frailty using two alternative widely accepted models. The first of these, the Rockwood Frailty Index (FI), is based on a cumulative deficit model of dysfunctions and impairments involving multiple systems and levels [23], and is expressed as a continuous variable. The second model, the Fried frailty status, is based on a purely physical phenotype, comprising five components (weight loss, weakness, slowness, exhaustion, and reduced physical activity), and is expressed as a categorical variable (0 = robust, 1–2 = pre-frail, and 3–5 = frail) [24]. A detailed distribution of these physical components and their combinations in our cohort are presented in Figure 1A. Among the 76 Chinese elderly that took part in the study, 41% (n = 31) were considered robust, 46% (n = 35) were pre-frail, and 13% (n = 10) were frail. Of the forty-five older adults (59%) suffering from one or more components of frailty, weakness (n = 27) and slowness (n = 21) were the most common, followed by low physical activity (n = 12) and exhaustion (n = 11), and lastly weight loss (n = 9).

The predictive validity of both models of frailty has been demonstrated in previous investigations [23–26] and is supported in this study sample. Although not based on identical biological constructs, the Frailty Index and Fried's categorical physical frailty status were highly concordant (Spearman's rho = 0.56, *p* < 0.001). Furthermore, both models were comparable in predicting basic Activities of Daily Living or Instrumental Activities of Daily Living (ADL-IADL) dependency; poor physical health, defined by the lowest tertile of Short Form 12 Physical Component Summary (SF12-PCS); and risk of hospitalization in the past year, with areas under the receiver operating characteristic (ROC) curve of 0.76 to 0.91 (Figure 1B).

Detailed demographic and clinical characteristics of the study participants, categorized according to the FI and frailty measure, are listed in Table 1. Frailty status, as determined by both the FI and frailty measure, was significantly associated with lower formal education level; impaired cognitive function measured by the Mini-Mental State Examination (MMSE); impaired balance and gait; reduced forced expiratory volume in 1 second (FEV1)/ forced vital capacity (FVC) ratio; increased depressive symptoms; increased monocyte numbers; and an increased proportion of individuals with raised fasting plasma glucose (FPG), raised high-density lipoprotein cholesterol (HDL-C) levels, and metabolic syndrome. No association was detected between frailty status and the proportion of individuals with dementia, as assessed by the Informant Questionnaire on Cognitive Decline in the Elderly (IQCODE), the Clinical Dementia Rating (CDR), self-report, or carrier status of ApoE e4, a genetic predisposing factor for dementia (*p* > 0.05). No difference in kidney function, assessed by estimated glomerular filtration rate (eGFR), was observed among the participants.

**Table 1:** Clinical and laboratory markers in the elderly according to the Fried frailty status

	Frailty Index		Fried Frailty Status			F/χ2	*p*
	*r*	*p*	Frail (*n* = 10)	Pre-frail (*n* = 35)	Robust (*n* = 31)		
Gender (female)	0.142	0.219	9 (90.0)	18 (51.4)	18 (58.1)	4.820	0.090
Age (years)	0.282	0.013	71.30 ± 8.93	69.31 ± 8.36	66.45 ± 7.23	1.809	0.171
Formal education (secondary and above)	0.041	0.724	0 (0.0)	7 (20.0)	13 (41.9)	10.231	0.037
MMSE score	−0.243	0.033	24.70 ± 3.34^*^	26.43 ± 4.24	28.13 ± 3.29	3.628	0.031
Dementia	0.242	0.075	2 (28.6)	4 (17.4)	2 (8.3)	1.970	0.374
ApoE-ε4 carrier	−0.080	0.487	1 (10.0)	6 (17.1)	1 (3.2)	3.384	0.184
CHS frailty score	0.562	<0.001	3.00 ± 0.00^***+++^	1.40 ± 0.50^***^	0.00 ± 0.00	332.33	<0.001
Frailty Index	1.000	<0.001	0.37 ± 0.04^***++^	0.31 ± 0.06^***^	0.26 ± 0.04	19.242	<0.001
Number of cumulative deficits	1.000	<0.001	16.67 ± 2.00^***++^	13.94 ± 2.61^***^	11.71 ± 1.83	19.243	<0.001
Number of doctor visit	0.686	<0.001	12.40 ± 7.58^*^	8.89 ± 9.24	4.61 ± 5.77	4.670	0.012
POMA balance	−0.282	0.013	14.67 ± 3.32^*+^	15.89 ± 0.53	15.93 ± 0.25	4.361	0.016
POMA gait	−0.188	0.102	10.22 ± 2.95^**^	11.57 ± 1.72	12.00 ± 0.00	4.668	0.012
Number of ADL disability	0.296	0.009	0.30 ± 0.48^***++^	0.03 ± 0.17	0.00 ± 0.00	8.533	<0.001
Number of IADL disability	0.103	0.374	8.90 ± 1.52	8.46 ± 1.65	8.00 ± 0.00	2.311	0.106
Any disability in ADL or IADL	0.282	0.013	4 (40.0)	4 (11.4)	0 (0.0)	12.901	0.002
Depressive symptom	0.601	<0.001	7 (70.0)	17 (48.6)	13 (41.9)	2.384	0.304
FEV1/FVC	−0.163	0.189	49.37 ± 30.59^*^	68.38 ± 25.90	72.85 ± 14.56	3.470	0.037
eGFR (ng/ml)	−0.068	0.575	66.78 ± 7.84	68.81 ± 12.03	72.35 ± 10.28	1.237	0.297
Monocyte (%)	0.077	0.510	7.05 ± 1.18	8.44 ± 2.29^*^	7.04 ± 1.44	5.267	0.007
Lymphocyte (%)	−0.198	0.086	33.12 ± 10.21	32.30 ± 7.76	35.19 ± 8.69	0.958	0.389
WBC (×10^9^/L)	0.111	0.339	6.49 ± 1.65	6.94 ± 1.77	6.96 ± 2.87	0.179	0.837
Hemoglobin (g/dL)	−0.295	0.009	12.07 ± 1.24	13.24 ± 1.82	13.42 ± 1.63	2.515	0.088
Raised TG	0.099	0.394	7 (70.0)	16 (45.7)	12 (38.7)	2.983	0.225
Raised FPG	0.347	0.002	6 (60.0)	9 (25.7)	6 (19.4)	6.366	0.041
Reduced HDL	0.302	0.008	6 (60.0)	7 (20.0)	7 (22.6)	6.794	0.033
Metabolic syndrome	0.631	<0.001	8 (80.0)	21 (60.0)	12 (38.7)	6.145	0.046
							

Data are presented as mean ± SD or n (%). MMSE = mini–mental state examination; ApoE = Apolipoprotein E; CHS = Cardiovascular Health Study; SF-12: 12-item Short Form Health Survey; POMA = Tinetti Performance Oriented Mobility Assessment; ADL = Activities of Daily Living; IADL = Instrumental Activities of Daily Living; FEV1 = forced expiratory volume in 1 second; FVC = forced vital capacity; eGFR = estimated glomerular filtration rate; WBC = white blood cell; TG = triglyceride; FPG = fasting plasma glucose; HDL = high-density lipoprotein. Reduced HDL: < 1.03 mmol/L in males and < 1.29 mmol/L in females; Raised FPG: > 5.6 mmol/L. *** *p* < 0.001, ** *p* < 0.01, * *p* < 0.05 vs. the robust group; +++ *p* < 0.001, ++ *p* < 0.01, + *p* < 0.05 vs. the pre-fail group.

### Cytokines, chemokines, and their receptors

**Figure 2:**
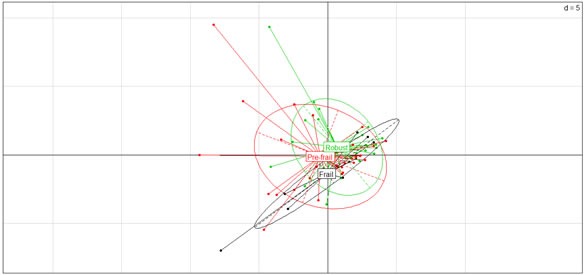
Visualized data structure of serological biomarkers in elderly according to frailty status Principal Component Analysis (PCA) was performed using the ade4 package of R. Identified components are shown in frail (black), pre-frail (red), and robust (green) groups.

The levels of 89 candidate inflammatory markers of aging (list shown in Supplementary Table 1) were measured in the blood of study participants. Principal component analysis (PCA) of the data in frail (black), pre-frail (red) and robust (green) subjects is shown in Figure 2. The first five extracted components explain 52.66% of the total variance of predictor variables. An exploratory stepwise linear regression technique was employed to identify significant sets of serological biomarkers that predict frailty. Candidate variables were entered at *p* < 0.10, and final sets were retained at *p* < 0.05. This analysis identified positive associations of the Frailty Index with levels of soluble glycoprotein 130 (sgp130, *p* < 0.001), I-309 (*p* = 0.010), B-cell attracting chemokine 1 (BCA-1, *p* < 0.001), RANTES (*p* = 0.013)), and leptin (*p* = 0.015). Monocyte chemoattractant protein-1 (MCP-1) was negatively associated with the Frailty Index (*p* > 0.05) (Table 2). In addition, a significant association of five serological markers (Table 3) with the Fried frailty status was detected in the final selected models of stepwise ordinal regression analysis, including the aforementioned sgp130 (p = 0.017), I-309 (p = 0.009), and MCP-1 (p = 0.008), as well as IL-6R (p = 0.024) and IL-2Rα (p = 0.038). No significant association with the FI or Fried's frailty status was observed among the other 81 candidate serological biomarkers.

**Table 2:** Serological biomarkers and immune cell phenotypes predicting Frailty Index (stepwise linear regression)

	Concentrations				Frailty Index			
	Mean	± SD	Range	B	SE	β	t	p
Cytokines/ Chemokines								
sgp130 (ng/ml)	154.86	37.23	62.50-261.00	0.001	0.000	0.372	3.885	<0.001
I-309 (pg/ml)	3.18	2.86	0.38-16.27	0.006	0.002	0.266	2.657	0.010
MCP-1 (pg/ml)	238.87	62.66	49.53-407.8	0.000	0.000	−0.345	−3.365	0.001
BCA-1 (pg/ml)	15.59	52.09	2.52-392.6	0.000	0.000	0.361	3.810	<0.001
RANTES (pg/ml)	1166.8	499.4	153.4-2649.9	0.000	0.000	0.251	2.560	0.013
Leptin (ng/ml)	7.23	8.50	0.70-50.85	0.002	0.001	0.233	2.509	0.015
α/β T Cell								
CD3+	63.31	10.48	40.90-83.5	−0.002	0.000	−0.333	−6.755	<0.001
CD27+CD45RA- %CD4	48.63	13.05	20.10-74.5	−0.002	0.000	−0.442	−6.797	<0.001
CD4/CD8 ratio of CD28+	93.56	6.03	72.90-100.0	0.021	0.003	0.377	6.515	<0.001
CD45RA+ %CD8	30.82	16.05	3.20-73.7	−0.001	0.000	−0.330	−5.656	<0.001
γ/δ T Cell								
CD27+	47.68	20.50	7.20-87.30	−0.001	0.000	−0.195	−3.763	<0.001
V/δ2+ IFN-γ+TNF-α-	4.89	1.98	0.91-11.60	0.008	0.001	0.327	6.184	<0.001
V/δ2- IFN-γ-TNF-α+	25.10	8.50	9.04-50.10	0.002	0.000	0.278	4.491	<0.001
B cell								
Exhausted	9.50	5.27	2.70-27.50	0.007	0.001	0.585	9.392	<0.001
CD24-CD38-	4.54	4.09	1.00-25.40	−0.010	0.002	−0.491	−6.034	<0.001
CD24-CD38+	8.94	6.17	0.70-32.00	0.006	0.001	0.597	8.976	<0.001
CD24+CD38+	50.46	13.82	2.20-75.50	0.001	0.001	0.251	2.235	0.030
CD24++CD38+	9.26	5.12	1.30-23.20	0.005	0.001	0.458	5.325	<0.001
IgM+IgD-	7.38	5.38	1.90-35.00	−0.004	0.001	−0.356	−6.522	<0.001
APC								
CD14+CD16+ %CD45+	1.86	1.11	0.01-5.38	0.034	0.003	0.595	9.609	<0.001

Frailty Index was derived as the count of observed clinical deficits relative to 45 possible deficits that included self-rated health, fall, hearing impairment, unintended weight loss, BMI < 18.5 or BMI > 30, low knee extension, slow waking speed, polypharmacy, bowels (preceding week), bladder (preceding week), grooming (preceding 24–48 h), toilet use, feeding, transfer (from bed to chair and back), mobility (about the house), dressing, stairs, bathing, using telephone, travelling, shopping, preparing meals, housework, doing laundry, taking medicine, managing money, hypertension, diabetes, stroke, heart disease, history of eye problem, history of kidney failure, history of asthma, history of COPD, history of tuberculosis, history of arthritis, history of osteoporosis, history of hip fracture, history of Alzheimer's disease, history of Parkinson's disease, depression, history of gastrointestinal problem, history of thyroid problem, history of cancer, other mental disorders.

### Immune cell phenotypes

We performed exploratory analyses of the association of frailty with immune cell phenotypes and markers of senescence in immune cell subsets. In α/β T cell subsets, the Frailty Index was positively associated with an altered CD4/CD8 ratio in CD28-expressing T cells, and inversely associated with the frequency of CD3+, central memory (CD27+CD45RA-) CD4+ cells, and CD45RA+CD8+ cells. The Fried frailty status, analyzed as an ordinal variable, was predicted by the frequency of terminal effector CD8+ T cells (T_TE_). In γ/δ T cells, the FI was associated with a decreased frequency of CD27 expression, increased expression of IFNγ+TNFα- in highly differentiated γ/δ2+ cells, and increased expression of IFNγ-TNFα+ in γ/δ2- CD3+ T cells. Frailty and pre-frailty categories were associated with a decreased frequency of CD27 and CD57. Both the FI and the Fried frailty status were associated with increased frequencies of CD38+ B cells. The FI also identified an association with increased levels of exhausted B cells and decreased frequencies of IgD+ B cells. In the myeloid compartment, a reduction in frequency of the CD14+CD16+ inflammatory monocyte subset was also identified as a frailty-associated phenotype (Tables 2-3).

**Table 3:** Serological biomarkers and immune parameters predicting Fried frailty status by stepwise ordinal regression

	Concentrations			Fried Frailty Status			
	Mean	± SD	Range	Estimate	SE	Wald	*p*
							
Cytokines/ Chemokines							
sgp130 (ng/ml)	154.86	37.23	62.50-261.00	0.019	0.008	5.739	0.017
I-309 (pg/ml)	3.18	2.86	0.38-16.27	0.271	0.103	6.911	0.009
MCP-1 (pg/ml)	238.87	62.66	49.53-407.8	−0.013	0.005	6.940	0.008
IL-6R (ng/ml)	16.20	4.40	6.44-26.71	−0.147	0.065	5.081	0.024
IL-2Ra (pg/ml)	626.85	266.76	204.35-1491.7	2.339	1.128	4.299	0.038
							
α/β T Cell							
CD27-CD45RA+ %CD8	30.82	16.05	3.20-73.7	0.032	0.015	4.591	0.032
							
γ/δ T Cell							
CD27+	47.68	20.50	7.20-87.30	−0.081	0.024	10.906	001
CD57+	43.50	20.57	10.80-86.40	−0.057	0.021	7.287	0.007
							
B cell							
CD24-CD38-	4.54	4.09	1.00-25.40	−0.318	0.118	7.329	0.007
CD24-CD38+	8.94	6.17	0.70-32.00	0.093	0.043	4.643	0.031
							
APC							
CD14+CD16+ %CD45+	1.86	1.11	0.01-5.38	0.476	0.224	4.510	0.034
							

*p < 0.05, **p < 0.01, ***p < 0.001 vs the robust group; #*p* < 0.05 *vs* the pre-frail group.

**Figure 3:**
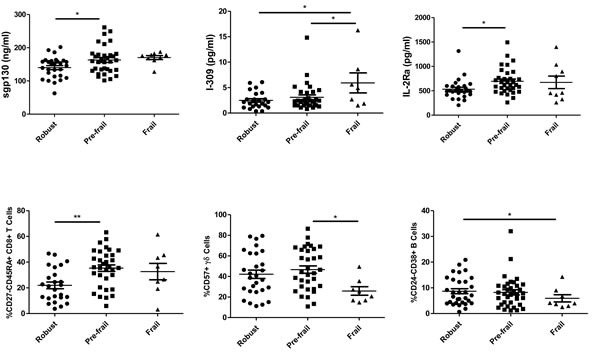
Comparison of selected predictors by stepwise ordinal regression in different frailty status groups The parameters include sgp130, I-309, IL-2Ra, CD27-CD45RA+ CD8+ T cells, CD57+ γ/δ T cells, and CD24-CD38+ B cells. ANOVA was performed followed by post-hoc pairwise comparisons using Bonferroni correction. Data are shown as mean ± SEM.

Figure 3 shows a comparison of the selected predictors by stepwise ordinal regression across the different frailty status groups. Pre-frail elderly exhibited significantly higher levels of sgp130 and IL-2Rα, and an increased frequency of CD45RA+CD8+ cells compared with their robust counterparts. Higher I-309 levels were observed in the frail group compared with the robust and the pre-frail groups. Frail elderly also exhibited a decreased frequency of CD57 in γ/δ T cells compared with pre-frail elderly, and a decreased frequency of CD24-CD38+ B cells compared with the robust elderly.

## DISCUSSION

The present study identified a number of serological biomarkers (sgp130, IL-2Rα, I-309, MCP-1, BCA 1, RANTES, leptin, and IL-6R) that are associated with frailty in the elderly, supporting the hypothesis that chronic systemic inflammation is involved in the biology of frailty [27, 28].

Previous studies have reported higher circulating IL-6 in frail elderly compared with their non-frail counterparts, associated with low walking speed, poor muscle strength, poor lower extremity performance, and anemia [29, 30]. The activity of circulating IL-6 is highly regulated by its free-circulating soluble receptors sgp130 and IL-6R, which are less prone to fluctuation than IL-6 [31] and are therefore considered more robust markers for the measurement of overall IL-6 activity. The present study detected a significant association of raised sgp130 and reduced IL-6R levels with frailty, suggesting that IL-6 receptors and the IL-6:sIL-6R:sgp130 complex may be involved in the development of frailty among the elderly. sIL-6R is negatively associated with bone mineral density, and IL-6 promotor polymorphism independently predicts bone mineral density and peak bone mass [32, 33]. Daily administration of low dose IL-6 to rhesus monkeys for 1 month lead to 10% body weight loss (primarily lean body weight), decreased albumin and cholesterol levels, increased CRP and alkaline phosphatase levels, and the animals became osteopenic and anemic [34]. In the central nervous system, IL-6 and IL-6R promote chronic inflammation and contribute to neurodegeneration and the development of Alzheimer's disease [35]. Thus, we could speculate that the effects of the IL-6 system on frailty, especially sarcopenia, may involve their contribution to lean body mass decrease, bone mineral density decrease, anemia, thrombocytosis, cholesterol and albumin decrease, insulin resistance, and cognitive impairment. The role of sgp130 in aging and frailty remains unclear. While sgp130 levels were significantly correlated with age in a study of n = 1010 elderly Italians [36], another study reported no association between plasma sgp130 concentration and functional dependence in 120 institutionalized elderly individuals as measured by the Barthel Index (BI) and Katz Index (KI) [37]. In addition, long-term exercise training in elderly men was found to improve frailty-related functional decline, but sgp130 levels were unchanged [37].

Consistent with previous findings of altered levels of the inflammatory chemokines, MCP-1 (CCL2) [38, 39] and RANTES [40, 41] during aging, the present study identified a positive association of MCP-1 and RANTES with frailty. Furthermore, an association with a number of chemokines and immune receptors not previously related to frailty were identified. BCA-1, also known as CXCL13 or BLC, is a potent B cell homing chemokine, which competes with IP-10 for binding to the CXCR3 receptor, and attenuates the calcium flux induced by IP-10 in B lymphocytes [42, 43]. I-309 (CCL-1) is a chemotactic cytokine that attracts monocytes, NK cells, and immature B cells and dendritic cells via interaction with CCR8. I-309 functions as an HIV co-receptor and is also involved in tumor progression [44, 45]. The sIL-2Rα (sCD25) acts as a decoy receptor to block the activity induced by its ligand IL-2, a pro-inflammatory cytokine that promotes T cell activation and proliferation [46]. Although frailty has been previously associated with reduced production of IL-2 [47], we show for the first time to our knowledge, that sIL-2Rα is an independent risk factor for frailty. These findings support the notion that changes in chemokine and cytokine-mediated inflammatory responses and cellular migration are associated with the development of frailty in the elderly. Because frail individuals are considered at-risk for various conditions, we can hypothesize that altered inflammatory and chemokine levels may reflect different requirements for cell activation, migration, and regulation. The observed imbalance in inflammation may be a contributing factor to individual components of frailty (e.g., sarcopenia), but may also be a consequence (e.g., weight loss).

In addition to the soluble factors associated with frailty identified here, our data also supports a link between specific immune cell phenotypes and physical frailty [12, 48]. We observed that a high Frailty Index was negatively predicted by the frequency of CD3+ T cells, CD45RA+ in CD8+ cells, and central memory CD4+ cells, and was positively predicted by an altered CD4/CD8 ratio in CD28+ T cells. Frail and pre-frail groups compared with the robust group were predicted by the frequency of terminal effector CD8+ T cells. The marked decline in CD28 expression in CD8+ T cells compared with CD4+ T cells may relate to the age-related decline in mitogen-induced T cell induction, most likely an adaptation to deal with chronic stimulation and inflammation occurring in the elderly [49].

Our results support a hypothetical involvement of γ/δ T cells in physical frailty, which display a different pattern of association from α/β T cells. We measured the expression of the major markers of α/β T cell aging, including CD27, CD28 (co-stimulatory receptors), and CD57 (marker of senescence in α/β T cells), in the γ/δ T cell population, and found that a decreased frequency of CD27 and CD57 expression was associated with frailty in the elderly. We previously showed that aging is not associated with differential expression of these two markers in γ/δ T cells [50], suggesting that this is specific to the frailty-associated process. Because γ/δ T cells secrete the pro-inflammatory Th1-like cytokines, IFN-γ and TNF-α [50], the increased frequency of IFN-γ+ and TNF-α+ cells may be a hallmark of altered responsiveness to persistent stress and contribute to the inflammatory profile in the frail category.

Changes in the humoral immune response that occur with aging are known to be mediated by B cells, leading to susceptibility to disease, poor responsiveness to vaccination, and increased risk of cancer. Our study indicates that frailty is associated with an increase in the levels of exhausted B cells (CD19+IgD-CD27-) and CD38+ B cells, as well as decreased IgD+ B cells. This is in line with recent human and animal studies showing B cell defects in the aging process [51].

Our finding that DCs were not associated with frailty is consistent with previous studies showing that DCs are relatively stable and there is no significant age-related change in phenotype or function [19, 52]. A decreased frequency of CD14+CD16+ monocytes has been reported in the advanced-age frail elderly [22]. In contrast, we observed an increased frequency of this population. This discrepancy may be related to the age and health status of the participants in these studies. Verschoor et al. [22] studied elderly people aged 81-100 years old, recruited from nursing homes; while our study included community-dwelling elderly people aged 55 and above (mean age: 68 ± 8 years). The advanced age of participants in the study by Verschoor et al. may “pre-select” those with longer lifespans and improved health status, including reduced levels of CD14+CD16+ cells, which can contribute to persistent secretion of pro-inflammatory cytokines, such as TNF-α, and the subsequent onset or development of frailty.

CMV status, identified as part of the Immune Risk Profile in a Swedish elderly population aged 85 and over [9,10], failed to enter the predictive regression models in this study. This is most likely a result of the very high CMV prevalence in Singapore (99%) [53]. Considering the variability of CMV prevalence globally, the role of CMV in elderly frailty warrants further investigation in other countries.

In this exploratory study, we made use of high-throughput assays and comprehensive immunological phenotypes to investigate the involvement of circulating biomarkers and immunosenescence in age-related frailty. We adopted two widely accepted definitions of frailty, which although not based on identical biological constructs include considerable overlap. The shared associations of frailty in both models with certain markers increase confidence in these findings. Given the more restricted physical construct of the Fried physical frailty phenotype, it is perhaps unsurprising that it was significantly associated with fewer serological biomarkers and phenotypes.

While our studies support a relationship between changes in inflammation and the cellular immune system with the development of frailty, it should be noted that the cross-sectional design of this study limits its ability to infer causal relationships. Future prospective studies, with larger sample sizes, should be conducted to establish “immune frailty” as a prognostic indicator of physical frailty syndrome.

## MATERIALS AND METHODS

### Study design and participants

Study participants were recruited from the Singapore Longitudinal Aging Study Wave 2 (SLAS-2), which is an ongoing population-based cohort study of aging and health among Chinese older adults aged 55 and above. Older adults who were residents in five adjoining districts in Singapore were identified by door-to-door census, and invited to participate in the study. Participants had a mean age of 68.41 years (SD: 8.08; range: 55-84). Among them, 59.21% were females, and 27.63% were educated at secondary level and above. Participants underwent 5–6 interview sessions, performance-based testing, and venesection by trained research nurses for an extensive measurement of demographic, neurocognitive, medical, psychosocial, and biological variables. The study excluded those who were physically or mentally unable to give informed consent or participate. The study was approved by the National University of Singapore Institutional Review Board, and all participants provided written informed consent (response rate: 78%).

Cognitive function was measured using Mini-mental State Examination (MMSE) validated in local Singaporean elderly [54]. Presence of medical disorders was ascertained by the subjects' self-report of a doctor's diagnosis and treatment, with the examination of their medication packages. The total number of medical disorders was calculated and number of hospitalizations in the past year was recorded. Presence of depressive symptoms was similarly determined. Short Form 12 Physical Component Summary (SF12-PCS) [55] was used to measure participants' physical health. Functional dependency was defined based on self-reported difficulty and requiring help on one or more IADL or basic ADL activities [56]. Participants' static and dynamic balance abilities were assessed using the Performance Oriented Mobility Assessment (POMA) [57]. Pulmonary function was assessed by the ratio of the FEV1 to FVC. Fasting venous blood was collected to test the levels of hemoglobin, TG, eGFR, glucose, HDL, and to perform lymphocyte, monocyte, and white blood cell (WBC) counts.

### Physical frailty measurements

The Fried physical frailty status was assessed based on the five syndrome components proposed and validated in the Cardiovascular Health Study [24]. Involuntary or unintentional weight loss was defined as a body mass index (BMI) below 18.5 kg/m^2^ and/or unintentional weight loss of more than 10 pounds (4.5 kg) in the past 6 months. Slowness was classified as the lowest quintile values (stratified for gender and height) in the average of two measurements of the 6-m fast gait speed test. Weakness was measured by dominant knee extension, and participants in the lowest quintile of a gender- and BMI-adjusted average value from three trials were defined as weak. Exhaustion was denoted as a score of < 10/15 on the vitality domain in the SF-12. Physical activities were assessed using self-reported time (in hours) spent daily doing light, moderate and vigorous activities. Participants were defined as low activity if the total amount of time they spent on performing moderate to vigorous activities per week was in the gender-specific lowest quintile. A participant with three or more components was grouped as frail, 1–2 components as pre-frail, and none of the components as robust.

Based on Rockwood's accumulative deficit model for frailty [23], a continuous variable of frailty index (FI) was calculated as the number of cumulative deficits out of 45 multisystem risk factors, which include self-rated health, fall, hearing impairment, unintended weight loss, BMI < 18.5 or BMI > 30, low knee extension, slow walking speed, polypharmacy, bowels (preceding week), bladder (preceding week), grooming (preceding 24–48 h), toilet use, feeding, transfer (from bed to chair and back), mobility (about the house), dressing, stairs, bathing, using the telephone, travelling, shopping, preparing meals, housework, doing laundry, taking medicine, managing money, hypertension, diabetes, stroke, heart disease, and a history of eye problems, kidney failure, asthma, COPD, tuberculosis, arthritis, osteoporosis, hip fracture, Alzheimer's disease, Parkinson's disease, depression, gastrointestinal problem, thyroid problems, cancer, or other mental disorders. The FI ranges from 0 to 1, with a higher value indicating a higher risk for frailty.

### Serology

Venous blood was drawn from overnight fasting participants into BD Vacutainer® CPT™ Cell Preparation tubes with Sodium Citrate (BD Biosciences, San Jose, CA, USA). After centrifugation at 300 rcf for 20 min at room temperature, plasma and peripheral blood mononuclear cells (PBMCs) were isolated. Plasma was stored at −80°C before use. PBMCs were washed twice in PBS and cryopreserved in liquid nitrogen.

Serology tests on plasma samples were performed using high throughput Luminex technology (Millipore Corp., Billerica, MA). A total of 89 serological biomarkers were measured using the MILLIPLEX MAP Human Cytokine/Chemokine Magnetic Bead Panels I, II, and III (Millipore Corp., Billerica, MA) according to the manufacturer's instructions. After overnight incubation, the plates were read on a Flexmap 3D instrument (Luminex Corporation, Austin, Texas, USA), and data were analyzed using Bioplex Manager 6.0 software (Bio-Rad Laboratories, Hercules CA).

### Flow cytometry staining

PBMCs were cryopreserved in 90% fetal bovine serum (FBS) containing 10% DMSO. On the day of antibody staining, cells were thawed rapidly and washed extensively with PBS containing 10% FBS. Sample recovery was greater than 75% without loss of specific immune populations [58]. Viability was higher than 95% as tested by trypan blue exclusion. PBMCs were counted and allowed to rest for 2 h in FACS buffer (PBS containing 10% FBS, 5 mM EDTA, and 2 mM azide) before use (1 × 10^6^ PBMC/test).

T cells were labelled using the following markers: CD3-PE-Cy5.5 (Beckman Coulter, Brea, CA, USA), CD4-PECy7 (BioLegend, San Diego, USA), CD8-APC/Cy7 (BD Biosciences), pan-γ/δ-PE (BioLegend), CD45RA-eFluor605 (eBioscience, San Diego, CA, USA), CD57-Pacific Blue (BioLegend), CD28-PETexas Red (Beckman Coulter), CD27-APC (BioLegend), IFNg-FITC (eBioscience), and TNFα-PE (eBioscience).

B cells were labelled using CD19-Qdot655 (Invitrogen, Carlsbad, CA), CD20-FITC (BioLegend), CD27-V500 (BD Biosciences), CD38-PC5.5 (Beckman Coulter), CD21 PE-Cy7 (Beckman Coulter), HLA-DR eF605NC (eBioscience), CD10-PE/Cy5 (BioLegend), CD23-ECD (Beckman Coulter), CD24-PE (BioLegend), IgM-Pacific Blue (BioLegend), IgD-APC (BD Biosciences), and IgG-Alexa Fluor 700 (BD Biosciences).

DCs and monocyte were labelled using CD14-APC/Cy7 (BioLegend), CD16 PE-Cy7 (eBioscience), CD11c Alexa Fluor® 700 (eBioscience), HLA-DR-Pacific Blue (eBioscience), CD56 FITC (eBioscience), and CD123 PE (eBioscience).

All preparations included a Live/Dead marker (Invitrogen) to exclude false positive staining. Multicolor flow cytometry was performed in a BD five-laser LSRII flow cytometer (BD Biosciences). Flow cytometry data were analyzed using FlowJo (Treestar), FACSDiva (BD Biosciences), and Kaluza (Beckman Coulter).

### Statistical analysis

Data analysis was performed using IBM SPSS 22 software (IBM, USA) and R v3.2.3. We compared the differences in demographics, cognitive function, health status, clinical profile, and laboratory markers among the frail, pre-frail, and robust study participants using chi-squared tests and one-way ANOVA followed by Bonferroni's posthoc test for multiple comparisons. ROC curves were drawn to describe the area under curve (AUC), specificity, and sensitivity of frailty index and frailty status in predicting ADL-IADL dependency, lowest tertile of SF-12 PCS, and risk of hospitalization in the past week. Principal Component Analysis (PCA) was performed using the ade4 package of R to visualize the data structure of serological biomarkers. Stepwise linear regression and ordinal logistical regression analyses were performed to investigate the association of the levels of the 89 serological biomarkers and immunological phenotypes (α/β T cells, γ/δ T cells, B cells, DCs, and monocytes) with frailty index and categorical frailty status. The level of statistical significance was set at *p* < 0.05 with a two-sided distribution. ANOVA for selected predictors by stepwise ordinal regression was performed, followed by post-hoc pairwise comparisons using Bonferroni correction.

## SUPPLEMENTARY MATERIAL TABLE


