# Supplementary material for: Inflammatory and immune markers associated with physical frailty syndrome: findings from Singapore longitudinal aging studies
Source: Oncotarget. 2016 Apr 22;7(20):28783–95. doi: 10.18632/oncotarget.8939 (PMC5045356; doi:10.18632/oncotarget.8939)
Supplement: Supplementary file 1 [file oncotarget-07-28783-s001.pdf]

Inflammatory and immune markers associated with physical frailty syndrome: findings from Singapore longitudinal aging studies

Supplementary Material

Supplementary Table S1. List of 89 serological biomarkers that were assayed

IL-1RI	IL-1RII	IL-2Ra	IL-4R	IL-6R
RAGE	TNFRI	TNFRII	VEGFRI	VEGFR2
VEGFR3	sgp130	EGF	Eotaxin	Basic FGF
FGF-2	Flt-3L	Fractalkine	G-CSF	GM-CSF
GRO	sCD30	EGFR	IFN-α2	IFN γ
IL-12p70	IL-13	IL-15	IL-17	IL-1a
IL-1RA	IL-4	IL-5	IL-6	IL-7
IL-8	IL-9	IL-10	IP-10	MCP-1
MCP-3	MDC	MIP-1α	MIP-1β	PDGF-AA
PDGF-BB	RANTES	TGF-α	TNF-α	VEGF
CD40L	6CKine	BCA-1	CTACK	ENA-78
Eotaxin-2	Eotaxin-3	I-309	IL-16	IL-20
IL-23	IL-28a	LIF	MCP-2	MCP-4
MIP-1δ	SCF	SDF-1	TARC	TPO
TRAIL	Dengue IgG	CRP	CRP Interp	H. pylori IgG
H. pylori Interp	CH50	HSV2 IgG	HCV IgG	EBV (EBNA)
EBV (VCA)	HSV1 IgG	Chicken pox IgG	CMV	CMV IgM
CMV IgG	CMV (IgM)	CMV (IgA)	Leptin	
